# Integrated long non‐coding RNA analyses identify novel regulators of epithelial‐mesenchymal transition in the mouse model of pulmonary fibrosis

**DOI:** 10.1111/jcmm.12783

**Published:** 2016-01-29

**Authors:** Hao Sun, Junjie Chen, Wenyi Qian, Jiang Kang, Jun Wang, Lei Jiang, Li Qiao, Wei Chen, Jinsong Zhang

**Affiliations:** ^1^ Department of EmergencyThe First Affiliated Hospital with Nanjing Medical UniversityNanjingChina; ^2^ Department of Intensive Care UnitYixing People's HospitalYixingChina; ^3^ Key Lab of Modern ToxicologyMinistry of Education and Department of ToxicologySchool of Public HealthNanjing Medical UniversityNanjingChina; ^4^ Department of Hepatobiliary and Pancreatic SurgeryThe Second Affiliated HospitalZhejiang UniversityHangzhouChina

**Keywords:** pulmonary fibrosis, epithelial–mesenchymal transition, paraquat, long non‐coding RNA

## Abstract

Idiopathic pulmonary fibrosis (IPF) is a chronic fatal lung disease characterized by aberrant accumulation of fibroblast population and deposition of extra cellular matrix. Increasing evidence support that epithelial‐mesenchymal transition (EMT) of alveolar epithelial cells is a critical process in the pathogenesis of IPF. Although delivery of bleomycin to induce acute lung injury is the most well‐studied animal model of pulmonary fibrosis, there is considerable interest to pursue other models to understand the common and/or specific pathological mechanisms. In this study, we established a mouse model of pulmonary injury and progressive interstitial fibrosis *via* intraperitoneal injection of paraquat, a widely used herbicide known to cause pulmonary fibrosis in human. Using transcriptome sequencing and microarray analysis, we profiled expression of long non‐coding RNAs (lncRNAs) and identified 513 up‐regulated and 204 down‐regulated lncRNAs in paraquat‐induced fibrotic lung tissues. Gene ontology analysis revealed that the differentially expressed lncRNAs are implicated in cell differentiation, epithelium morphogenesis and wound healing, pathways closely associated with EMT. Furthermore, we identified the evolutionally conserved target genes of two up‐regulated lncRNAs, uc.77 and 2700086A05Rik, as *Zeb2* and *Hoxa3*, respectively, both of which are important modulators of EMT. Consistently, overexpression of uc.77 or 2700086A05Rik in human lung epithelial cells induced EMT as demonstrated by changes in gene and protein expression of various EMT markers and cell morphology. Collectively, our results uncovered a crucial role of lncRNA in the regulation of EMT during lung fibrosis and provide potential avenues for the discovery of novel molecular markers and therapeutic targets for IPF.

## Introduction

Idiopathic pulmonary fibrosis (IPF) is a chronic progressive disease of the lung interstitium with poor prognosis 1. Although the exact cause of IPF remains unknown, it is believed that IPF originates from repetitive micro‐injuries to the alveolar epithelium which may result from cigarette smoking, inhalation of dust, or occupation‐related exposure to hazardous chemicals 1, 2. Considerable efforts have been made to develop animal models of pulmonary fibrosis to understand the pathological mechanisms of IPF. Bleomycin, an anti‐cancer chemotherapeutic agent, is commonly used to induce acute lung damage and fibrosis in rodents 3. While studies using these models have generated important molecular insights, it remains unclear whether signalling pathways mediating bleomycin‐induced lung fibrosis are shared by other IPF risk factors. We reason that alternative animal models are critically needed to identify common and/or toxin‐specific mechanisms of lung fibrosis.

In particular, paraquat (1,1‐dimethyl‐4,4‐bipyridilium dichloride) is a widely used contact herbicide that can lead to development of fatal pulmonary fibrosis in human after accidental ingestion 4, 5. Despite extensive efforts to treat paraquat‐induced lung injury, the mortality remains high. While paraquat has been shown to induce pulmonary fibrosis in rodent models, the underlying molecular mechanism remains poorly understood and is likely to be distinct from that of bleomycin. For example, bleomycin is known to cause cell toxicity through initiating DNA damages, whereas paraquat has been shown to interfere with cellular redox network and elevate oxidative stress 4. Furthermore, a recent genome‐wide gene expression study revealed that a significant portion (>40%) of the paraquat‐induced transcriptional changes were not shared by bleomycin 6, highlighting the importance of understanding the paraquat‐specific pathological functions in lung fibrosis.

At late stage, the pathological hallmarks of IPF include massive accumulation of fibroblasts and myofibroblasts and deposition of extracellular matrix (ECM) in the alveoli 7. The abnormal genesis of fibrotic cells is thought to play critical roles in the destruction of alveolar architecture and the progressive impairment in lung function. Although their cellular origins remain to be fully elucidated, emerging evidence demonstrate that at least one‐third of the aberrantly accumulated fibroblasts are derived from lung epithelial cells *via* epithelial‐mesenchymal transition (EMT), a biological process that enables a polarized epithelial cell to obtain phenotypes of a mesenchymal cell 8, 9, 10. Epithelial‐mesenchymal transition frequently occurs during embryogenesis, organ development and wound healing and its misregulation has been shown to facilitate tumour metastasis 11. However, the molecular mechanisms of EMT in pulmonary fibrosis remain elusive 12.

Non‐coding RNAs (ncRNAs) are functional RNA molecules that are not translated into proteins 13. Depending on their length and functional characteristics, ncRNAs can be classified into several groups, including microRNA (19‐22 nucleotides) and long non‐coding RNA (>200 nucleotides) (lncRNA). Over the past decade, the biological importance of ncRNAs is increasingly appreciated. ncRNAs modulate a variety of cellular processes including gene transcription, imprinting, RNA splicing and protein translation and have been implicated in the regulation of various physiological pathways 14. Not surprisingly, mis‐regulation of ncRNAs are involved in the pathogenesis of many human diseases. Several microRNAs were shown to contribute to the initiation and progression of pulmonary fibrosis 15. In contrast, there is limited study on the expression profile of lncRNA during lung fibrosis and its functional impact has yet to be described.

In this study, we established a mouse model of paraquat‐induced pulmonary fibrosis. With this model, we performed transcriptome analyses and identified a set of lncRNAs that were differentially expressed between normal and fibrotic lung tissues. Functional analysis pointed to several EMT‐linked pathways that were highly enriched in differentially expressed lncRNAs. Furthermore, we predicted and used quantitative real‐time PCR (q‐RTPCR) analysis to confirm that two lncRNAs up‐regulated in paraquat‐treated lung tissues, uc.77 and 2700086A05Rik (05RiK), acted through *Zeb2* and *Hoxa3* genes respectively. Consistent with *Zeb2* and *Hoxa3* as key regulators of EMT, we found that transfecting human lung epithelial cells with uc.77 or 05Rik was sufficient to induce EMT. The novel findings that lncRNAs modulate EMT in the mouse model of lung fibrosis may open new avenues for the diagnosis and treatment of human IPF.

## Materials and methods

### Animals

BALB/c mice (8 weeks, male, 19.8 g ± 0.63 g) were provided by Experimental Animal Center of Nanjing Medical University, China. A total of 19 BALB/c mice were randomly divided into 2 groups (*n* = 9 in saline control group; *n* = 10 in paraquat‐treated group). Mice were housed on a constant 12 hr light/12 hr dark cycle in a temperature (22.2°C)‐controlled room and given *ad libitum* access to ordinary chow. These experiments were approved by the Animal Experiment Ethics Committee of Nanjing Medical University (Permission Number: IACUC‐14030122).

### Paraquat administration

Mice in the model group were administered 10 mg/kg paraquat (Cat. #: 36541; Sigma Chemical Co., St. Louis, MO, USA) dissolved in sterile saline *via* intraperitoneal injection (once per day for 5 days). The normal control group were administered an equal volume of saline at the same time. Mice were examined daily, bodyweights and survival rates were recorded daily. Lung tissues were harvested on the 12th day following the first treatment.

### Bronchoalveolar lavage

Mouse is cannulated with a blunt syringe catheter (20 G) after deeply anesthetized and tracheostomized. A clamp is placed on the left mainstem bronchus, and the right lung is lavaged with 3 × 0.5 ml 5 mM ethylenediaminetetraacetic acid in PBS. After centrifuged at 135 RCF (g) for 10 min., the supernatant fluid is then frozen at −80°C for subsequent analysis. The pellet is then resuspended in 1 ml PBS after red blood cells were lysed (Cat. #: R7757; Sigma Chemical Co.). 10 μl of the remaining fluid is then counted on a haemocytometer, and 150 μl is cyto‐spinned onto glass slides for Giemsa staining (Cat. #: 1.15213.0001; Hema‐Tek, Bayer Diagnostics, Leverkusen, Germany). Differential counts of neutrophils, lymphocytes and macrophages were determined using light microscopy (400× magnification, 100 cells counted) in a blinded manner.

### Haematoxylin and eosin and Masson's trichrome staining

Whole left lung tissue was fixed with 4% formalin overnight, dehydrated in 70% ethanol and cleared in xylene, 4 μm sections were prepared for assessing the general histological appearance by routine haematoxylin and eosin staining (Cat. #: 1.17081.1000; Hema‐Tek, Bayer Diagnostics). In addition, Masson's trichrome staining (Cat. #: 1.00485.0001; Hema‐Tek, Bayer Diagnostics) was used to demonstrate collagen deposition. The stained sections were viewed under the microscope (Zeiss Axioskop and Axiocam 5) at 100–400× magnification.

### Immunohistochemistry

Immunohistochemistry (IHC) for the detection of α‐SMA in formalin‐fixed paraffin‐embedded lung tissue was performed with an Elivision plus Polyer HRP IHC Kit (Cat. #: 9901; MXB, Nanjing, China) according to the manufacturer's instructions. The sections were incubated with a mouse anti‐α‐SMA antibody (Cat. #: bs‐0189R; Bioss, Beijing, China). The stained sections were viewed under the microscope (Zeiss Axioskop and Axiocam 5) at 100–400× magnification. The slides were scanned by a MIRAX Desk Digital Slide Scanner (Zeiss, Gottingen, Germany) and analysed by MIRAX Viewer (Zeiss).

### Immunofluorescence

For formalin‐fixed paraffin‐embedded tissues, slides were de‐waxed by washing with xylenes, rehydrated and blocked. For cultured cells, cells were seeded in chamber slides (Cat. #: C6807; Sigma Chemical Co.), fixed with 4% formaldehyde, permeabilized in 0.1% Triton‐X100, and blocked. Slides were then incubated with primary antibodies for α‐SMA (Cat. #: bs‐0189R; Bioss, Beijing, China), Vimentin (Cat. #: 5741; Cell Signaling Technology, Danvers, MA, USA), E‐cadherin (E‐cad, Cat. #: 14472; Cell Signaling Technology), or SP‐C (Cat. #: sc‐7706; Santa Cruz Biotechnology, Inc., Santa Cruz, CA, USA), for overnight at 4°C. Slides were then washed and stained with fluorophore‐conjugated secondary antibodies for 1 hr at room temperature. Cover slips were mounted using mounting medium (Cat. #: C9368; Sigma Chemical Co.), allowed to harden overnight at 4°C and then sealed. Images were acquired on Olympus IX70 (Japan) fluorescence microscope.

### Western blotting

Cells were harvested and lysed in 50 μl cell lysis buffer (Cat. #: 9803; Cell Signaling Technology) containing protease inhibitors (Cat. #: 11206893001; Sigma Chemical Co.). The protein concentration was quantified using the BCA Protein Kit (Cat. #: 23227; Thermo, Rockford, IL, USA). Cell lysates were separated by 10% SDS‐PAGE and proteins were transferred to polyvinylidene difluoride membranes (Cat. #: ISEQ00010; Millipore, Billerica, MA, USA). The membranes were then incubated with primary antibodies for Zeb2 (Cat. #: A300306; EarthOx LLC., San Francisco, CA, USA), Hoxa3 (Cat. #: sc‐28598; Santa Cruz Biotechnology, Inc.), α‐SMA (Cat. #:14968; Cell Signaling Technology), E‐cad (Cat. #: 14472; Cell Signaling Technology), N‐cadherin (Cat. #: 13116; Cell Signaling Technology), Vimentin (Cat. #: 5741; Cell Signaling Technology), and GAPDH (Cat.#: ab181602; Abcam, Cambridge, MA, USA) at 4°C overnight. The membranes were washed three times with TBS/T and then incubated with the appropriate HRP‐conjugated secondary antibodies for 1 hr at room temperature. Protein expression was detected by chemiluminescence (Cat. #: RPN2106; GE Healthcare, Piscataway, NJ, USA). Immunoreactive bands were quantified using the Bio‐Rad Gel Imaging System (Bio‐Rad, Berkeley, CA, USA).

### Hydroxyproline assay

The total collagen content in the right lungs was determined using hydroxyproline assay. The entire right lung tissues were homogenized with a tissue tearor (Kinematica AG, Littau, Lu, Switzerland), hydrolysed with 12 N HCl overnight at 100°C, oxidized by Citrate Acetate buffer, reconstituted with chloramine T solution [1.4% chloramine T (Cat. #: 31224) in 0.5 M sodium acetate (Cat. #: 42123) and 20% N‐propanol (Cat. #: 34871), all from Sigma‐Aldrich, St. Louis, MO, USA] and incubated for 20 min. at room temperature, and then reacted with Ehrlich's/pDMAB (1 M p‐diamethylaminobenzaldehyde [Cat. #: 39070; Sigma‐Aldrich] in 70% N‐propanol and 30% perchloric acid [Cat. #: 244252; Fluka, St. Louis, MO, USA]) and incubated at 65°C for 20 min. The standard cis‐4‐hydroxy‐1‐proline (Cat. #: H1637; Sigma‐Aldrich) is made up in sterile water to 4 mg/ml. The absorbance of the products was assessed colorimetrically at 550 nm in a microplate reader (BioTek Instruments Inc., Winooski, VT, USA). The amount of hydroxyproline was calculated as micrograms per millilitre lung sample by comparison to the standard curve.

### RNA extraction and quality control

Total RNA from lung tissues was isolated using TRIzol reagent (Cat. #: H1637; Invitrogen, Carlsbad, CA, USA) and the RNeasy Mini Kit (Cat. #: 74104; Qiagen, Hilden, Germany) according to the manufacturer's instructions. RNA quantification and quality check were performed with NanoDrop and Agilent 2100 Bioanalyzer (Agilent Technologies, Santa Clara, CA, USA) according to the manufacturer's instructions.

### Microarray analysis

LncRNA expression profiling was performed with service from Kangchen Biotech, Shanghai, China, based on the manufacturer's standard protocols with minor modifications. Briefly, mRNA was purified from 1 μg total RNA using mRNA‐ONLY^™^ Eukaryotic mRNA Isolation Kit (Cat. #: MOE51010; Epicentre, Madison, WI, USA). Then, each sample was amplified and transcribed into fluorescent cRNA along the entire length of the transcripts without 3′ bias utilizing a random priming method. The labelled cRNAs were hybridized onto the Mouse lncRNA Array v2.0 (Arraystar Inc., Rockville, MD, USA). After washing, the arrays were scanned by the Agilent Scanner G2505B. Agilent Array platform was employed for microarray analysis. Quantile normalization and subsequent data processing were performed with the GeneSpring GX v11.5.1 software package (Agilent Technologies).

### Quantitative real‐time RT‐PCR

Total RNA was isolated with the RNeasy Mini Kit (Cat. #: 74104; Qiagen, Valencia, CA, USA). 1 μg of RNA from each sample was reversely transcribed to cDNA using random hexamer primer with PrimeScript^™^ RT Master Mix (Cat. #: RR036A; TaKaRa, Otsu, Shiga, Japan). Primers for each lncRNA were designed according to Primer 3 (http://sourceforge.net/projects/primer3/) online and checked with Basic Local Alignment Search Tool of NCBI to ensure unique amplification product. Real‐time PCR was performed on an Applied Biosystems ViiA^™^ 7 Dx (Life Technologies, Carlsbad, CA, USA) using the SYBR green method according to the manufacturer's instructions. The PCR reaction conditions consisted of a denaturation step at 95°C for 30 sec., and then 40× PCR cycles at 95°C for 5 sec., 60°C for 34 sec. LncRNA and gene expression levels were quantified relative to the expression of GAPDH using an optimized comparative Ct (▵▵Ct) value method. All the primer sequences used were shown in Table S1. The 2^−∆∆Ct^ method was used to comparatively quantify the levels of mRNA.

### Gene Ontology analysis and KEGG pathway analysis

Using standard enrichment computation method, Gene Ontology (GO) and KEGG (Kyoto Encyclopedia of Genes and Genomes) pathway analyses were performed to determine the role of the closest genes to which lncRNAs preferentially located. The GO categories are derived from GO, which comprises three structured networks (Biological Process, Cellular Component and Molecular Function) of defined terms that describe gene product attributes 16. Fisher's exact test is used to find if there is more overlap between the differentially expressed lncRNA/gene list and the GO annotation list than what would be expected by chance. The *P* value denotes the significance of GO terms enrichment in the differentially expressed lncRNA/gene list. Pathway analysis is a functional analysis mapping genes to KEGG pathways. Base on the latest database, the pathway analysis was provided for differentially expressed lncRNAs. The *P* value (EASE‐score, Fisher‐*P* value or Hypergeometric‐*P* value) denotes the significance of the pathway correlated with the conditions.

### Bioinformatic analysis

The sequence conservation of lncRNAs and their associated protein‐coding genes were generated by using UCSC genome browser (http://genome.ucsc.edu/) and other databases as indicated. According to the relationship between lncRNAs and associated coding genes, lncRNAs can be roughly classified into four groups: cis‐antisense lncRNAs, intronic lncRNAs, promoter‐associated lncRNAs, and bi‐directional lncRNAs. In this study, various epigenetic phenomena were incorporated to facilitate identification of non‐coding regulatory elements.

### Cell lines and cell culture

Primary human bronchial epithelial cells and human lung epithelial A549 cells were purchased from the Beijing Institute for Cancer Research (Beijing, China). Human bronchial epithelial cells and human lung epithelial A549 cells were cultured in DMEM (Cat. #: 10566‐016; Thermo Fisher Scientific) with 10% foetal bovine serum. All cells were maintained at 37°C in 5% CO_2_ incubator.

### Plasmid construction and cell transfection

LncRNAs (uc.77 and 05Rik) were PCR amplified and cloned into eukaryotic expression pEX‐3 vector (Cat. #: C05003; GenePharma, Shanghai, China). For overexpression of lncRNAs, cells were transfected with plasmid and Lipofectamine 2000 (Cat. #: 11668027; Invitrogen) according to the manufacturer's instructions. Cells were harvested 2 days after transfection for the analysis of lncRNA/gene expression and cell morphology. For small‐interference RNA (siRNA) transfection, cells were transfected with ZEB2 siRNA (Cat. #: sc‐38641; Santa Cruz Biotechnology) using Lipofectamine 2000 according to the manufacturer's protocol.

### Statistical analysis

All statistical analyses were performed with SPSS17.0 software package (SPSS, Chicago, IL, USA). Differential expression levels of lncRNAs and their associated genes (uc.77/*Zeb2* and 05Rik/*Hoxa3* respectively) were determined by independent‐samples *t*‐test between two groups. Fisher's exact test was performed in GO and KEGG pathway analysis. Numerical data were presented as means ± S.D., and statistical significance was defined as *P* < 0.05.

### Data deposition

Array data were deposited at the Gene Expression Omnibus (National Center for Biotechnology Information) with the accession number GSE64851.

## Results

### Pathological characterization of paraquat‐induced pulmonary fibrosis in mice

**Figure 1:**
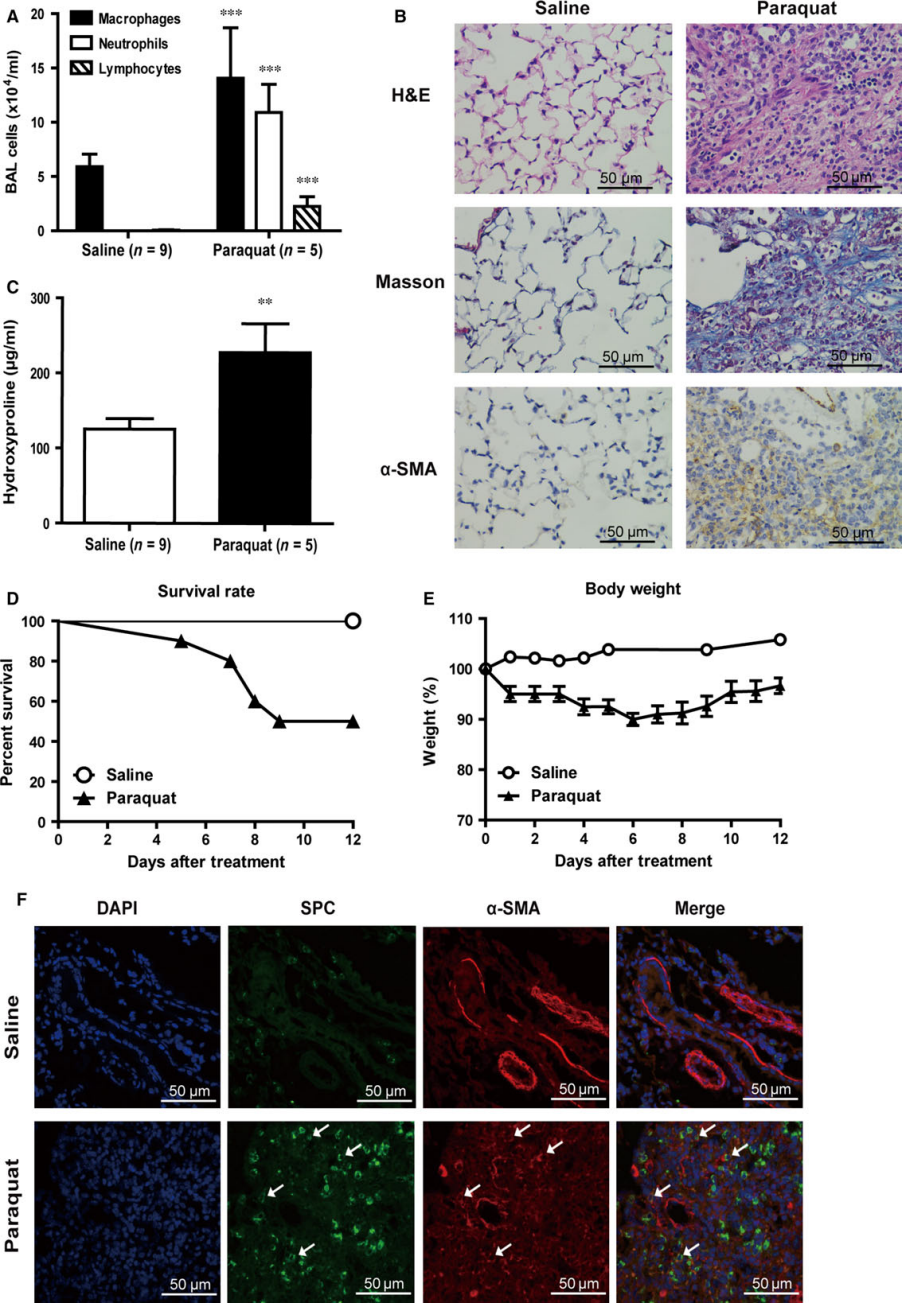
Establishing a mouse model of paraquat‐induced pulmonary fibrosis. (**A**) Quantification of bronchoalveolar lavage (BAL) cells after paraquat treatment. ***, *P* < 0.001 in comparison to saline‐treated control mice. (**B**) Haematoxylin and eosin stain, Masson's trichrome stain and IHC staining with α‐SMA antibody of interstitial lung tissues from control or paraquat‐treated mice. Images were taken at the magnification of ×400. Scale bar represents 50 μm. (**C**) Hydroxyproline levels in homogenates from the right lung of saline‐ or paraquat‐treated mice. **, *P* < 0.01 in comparison to saline‐treated control mice. (**D**) Percentage of survival of mice treated with paraquat or saline. (**E**) Daily measurement of mice weight following saline or paraquat treatment. (**F**) Immunofluorescence images showing staining of lung tissues from saline‐ or paraquat‐treated mice with DAPI (blue), or antibodies against SPC (green) or α‐SMA (red). Arrowheads point to cells positively stained for both SPC and α‐SMA. Images were taken at the magnification of ×400. Scale bar represents 50 μm.

We constructed a paraquat‐induced pulmonary fibrosis mouse model as described in the Materials and Methods. Paraquat‐treated mice showed significant increases in the total lavage cell counts and the percentage of neutrophils and lymphocytes compared to controls (*P* < 0.001; Fig. 1A). Haematoxylin and eosin staining and Masson's trichrome staining were used to assess the histopathological changes and collagen deposition after intraperitoneal injection of paraquat. Results revealed a well‐defined pulmonary structure with integral air‐blood barriers and low collagen levels in the control group. In contrast, in paraquat‐treated mice pulmonary tissue structure was distorted with severe damage to the air‐blood barrier. Significant thickening of the pulmonary inter‐alveolar septum and increased deposition of collagen fibres were noticed (Fig. 1B). We performed hydroxyproline assay to quantify the changes observed in the collagen content. The result indicated that hydroxyproline content in the whole right lung was significantly elevated in paraquat‐treated mice compared to saline‐treated controls (*P* < 0.01; Fig. 1C). In line with these findings, IHC staining with α‐SMA, a myofibroblast marker, revealed visible fibroblast accumulation after paraquat injection (Fig. 1B). In addition, we observed a decrease in mobility and a loss of bodyweight following paraquat treatment. Paraquat‐treated mice also exhibited significantly higher mortality rate than saline‐treated controls (Fig. 1D and E). Post‐mortem examination confirmed the major cause of death as pulmonary dysfunction because of either acute or chronic pulmonary injuries, and progressive interstitial fibrosis.

Previous studies using cell fate reporter mice concluded that a significant portion of the lung fibroblast population during fibrosis results from EMT of lung epithelial cells 8, 9. To confirm these findings in our paraquat‐induced pulmonary fibrosis model, we performed immunofluorescence (IF) co‐staining of the type II alveolar epithelial cells marker surfactant protein‐C (SPC) and an EMT marker α‐SMA. In paraquat‐treated but not normal lung tissues, we observed that a subset of cells were stained positively for both markers, suggesting that they were undergoing early stages of EMT (Fig. 1F).

### Expression profiling and analyses of lncRNA in paraquat‐induced lung fibrosis

**Figure 2:**
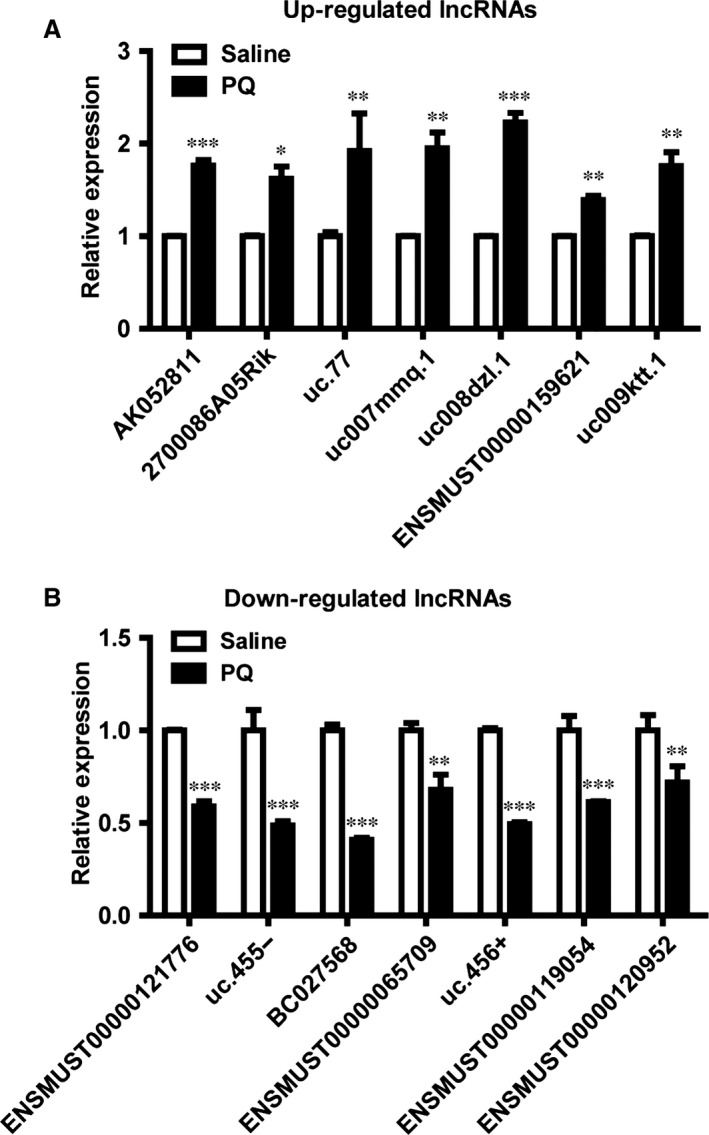
Expression profiling and validation of lung fibrosis‐associated lncRNA. (**A** and **B**) The differential expression of lncRNAs between saline‐ and paraquat‐treated mice was validated by q‐RTPCR. Error bar represents biological replicates (*n* = 3). *, *P* < 0.05, **, *P* < 0.01, ***, *P* < 0.001 in comparison to saline‐treated control mice.

Lung tissues from control or paraquat treatment group were subjected to microarray expression analysis of lncRNA and mRNA. A total of 31,423 lncRNAs and 25,376 coding mRNA transcripts were identified using the most authoritative databases such as Ensembl, UCSC Knowngenes, RefSeq and many related literatures (Fig. S1A). Scatter plot is adopted to visualize and assess the lncRNA or mRNA expression variation between control samples and paraquat‐treated fibrotic samples (Fig. S1B and C). With a cut‐off fold change of ≥5.0, we identified 513 up‐regulated and 204 down‐regulated lncRNAs in the paraquat‐induced fibrotic lung tissues compared to normal tissues (Table S2), suggesting that there was an extensive remodelling of the cellular lncRNA transcriptome during the fibrosis development upon paraquat treatment. Quantitative real‐time PCR was used to validate our microarray data, including seven up‐regulated lncRNAs (AK052811, 2700086A05Rik, uc.77, uc007mmq.1, uc008dzl.1, ENSMUST00000159621 and uc009ktt.1) and seven down‐regulated lncRNAs (ENSMUST00000121776, uc.455‐, BC027568, ENSMUST00000065709, uc.456+, ENSMUST00000119054 and ENSMUST00000120952) (Fig. 2A and B).

### GO and KEGG pathway analysis

**Figure 3:**
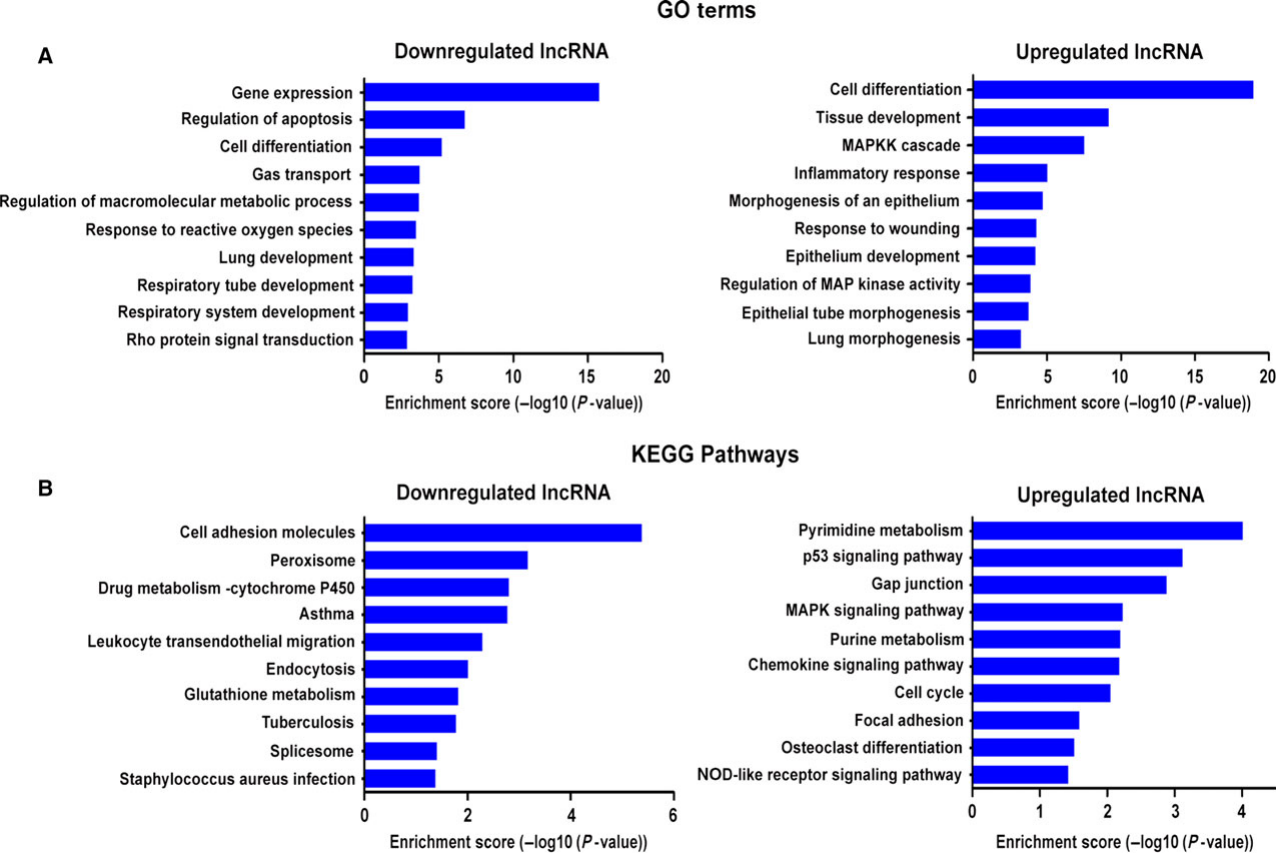
GO and KEGG pathway analysis. (**A**) The top 10 GO terms that are most statistically significantly enriched in up‐regulated (right) or down‐regulated (left) lncRNAs of paraquat‐treated mice. (**B**) The top 10 KEGG pathways that are most statistically significantly enriched in up‐regulated (right) or down‐regulated (left) lncRNAs of paraquat‐treated mice.

To obtain more insights into how mis‐expression of lncRNA may contribute to the pathogenesis of paraquat‐induced pulmonary fibrosis, we performed functional analysis of our microarray results. Gene Ontology analysis revealed that the most statistically significantly enriched functional terms in lncRNAs down‐regulated by paraquat treatment include gene expression, regulation of apoptosis and cell differentiation. The top enriched functional terms in up‐regulated lncRNAs include cell differentiation, tissue development and MAPKK cascade (Fig. 3A). In addition, KEGG pathway analysis was carried out for differentially expressed lncRNAs. Up to 19 pathways were enriched in up‐regulated lncRNAs including pyrimidine metabolism and p53 signalling pathway, while cell adhesion molecules and peroxisome were among the 34 pathways significantly associated with down‐regulated lncRNAs in paraquat‐treated lung tissues (Fig. 3B).

### Identification of potential lncRNAs involved in EMT

**Figure 4:**
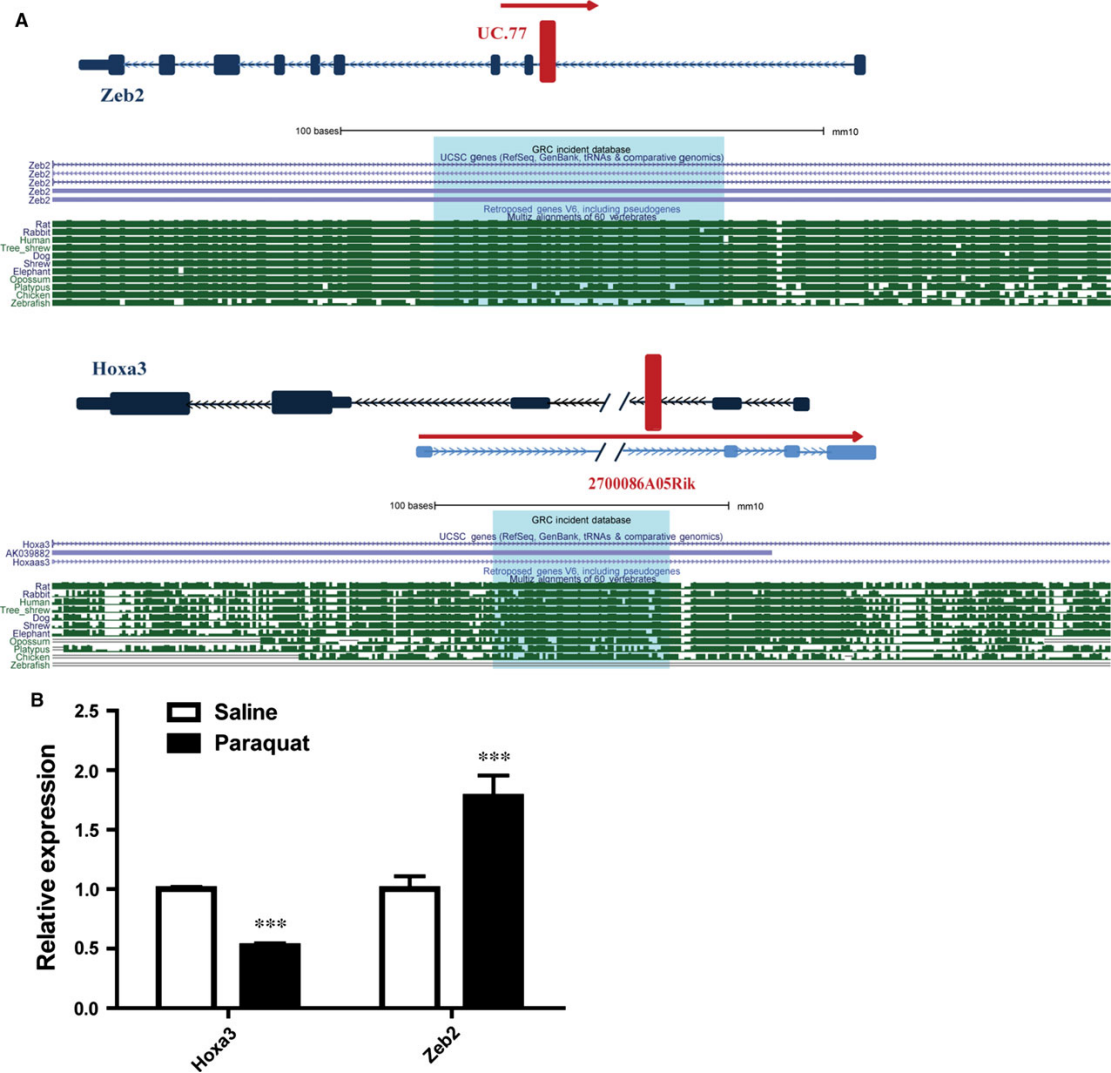
Target gene prediction for uc.77 and 05Rik. (**A**) Schematic representation of uc.77 and 05Rik loci. The red box shows the location of lncRNAs relative to *Zeb2* and *Hoxa3* genes respectively. Lower panels show the sequence alignments of the lncRNA loci in various species. (Download the browser graphic from: http://genome.ucsc.edu/cgi-bin/hgGateway). (**B**) Q‐RTPCR results showing the differential expression of *Hoxa3* and *Zeb2* in saline‐treated *versus* paraquat‐treated lung tissues. Error bar represents biological replicates (*n* = 4). ***, *P* < 0.001 in comparison to saline‐treated control mice).

Intriguingly, our functional analysis uncovered that several biological processes tightly associated with EMT, such as cell differentiation, tissue development, response to wounding and cell adhesion molecule, were among the top GO terms and KEGG pathways enriched in differentially expressed lncRNAs (Fig. 3). This result prompted us to investigate whether some of the mis‐expressed lncRNAs may regulate genes that play important roles in EMT. Interestingly, through querying UCSC genome browser (http://genome.ucsc.edu/) and NCBI database (http://blast.ncbi.nlm.nih.gov/), two up‐regulated lncRNAs: uc.77 and 2700086A05Rik (05Rik), were found to overlap with *Zeb2* and *Hoxa3* genes respectively. Zeb2 (zinc‐finger enhancer binding 2) is a transcription factor critical for the initiation of EMT 17. Notably, levels of Zeb2 are under the control of the miR‐200 family which is known to promote epithelial differentiation, while its regulation by lncRNA has not been reported. *Hoxa3* belongs to the homeobox family of genes which encode a highly conserved family of transcription factors that are important for morphogenesis and cell differentiation. In particular, *Hoxa3* has been implicated in the fate determination of mesenchymal tissues 18, 19. We found that uc.77 was located upstream of the intron of *Zeb2* gene, while 05Rik was located downstream of the intron of *Hoxa3* gene (Fig. 4A), raising the possibility that the expression of *Zeb2* and *Hoxa3* may be affected by uc.77 and 05Rik respectively. In agreement, *Zeb2* expression was up‐regulated in paraquat‐treated pulmonary tissues, while *Hoxa3* expression was down‐regulated (Fig. 4B). Importantly, the location and sequence of uc.77 and 05Rik are highly conserved (Fig. 4A), suggesting that these lncRNAs likely regulate the same target genes in a similar manner in human.

### uc.77 and 05Rik promote EMT in human lung epithelial cells

**Figure 5:**
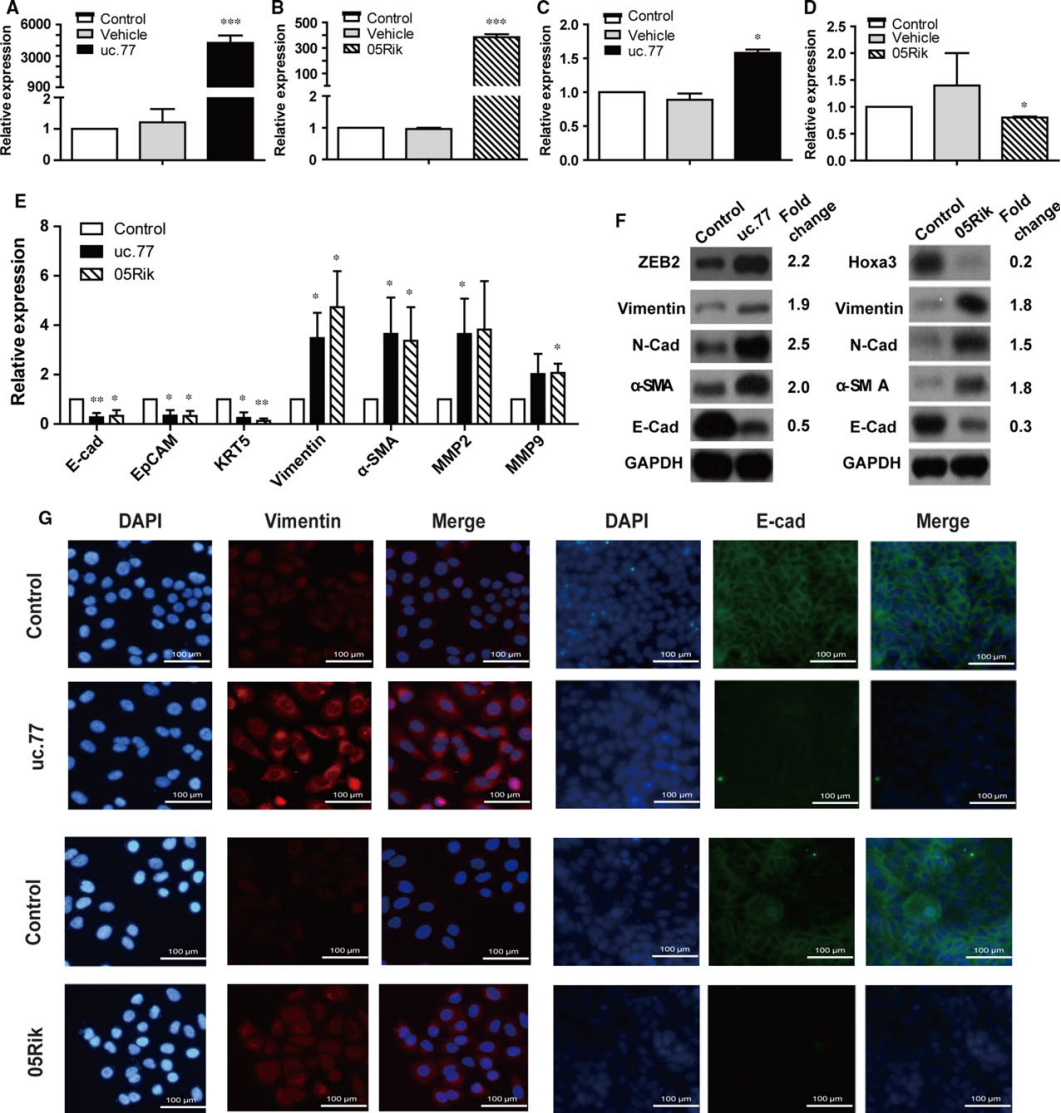
uc.77 and 05Rik lncRNAs regulate target gene expression and partially promote EMT. (**A** and **B**) Q‐RTPCR validation of the overexpression of uc.77 or 05Rik in A549 cells transfected with respective lncRNA. Error bar represents biological replicates (*n* = 3). ***, *P* < 0.001 in comparison to either mock‐transfected cells or cells transfected with control vector. (**C** and **D**) Q‐RTPCR measurement of the expression of *Zeb2* and *Hoxa3* in A549 cells transfected with uc.77 or 05Rik respectively. Error bar represents biological replicates (*n* = 3). *, *P* < 0.05 in comparison to either mock‐transfected cells or cells transfected with control vector. (**E**) Q‐RTPCR measurement of the expression of epithelial markers (E‐cadherin, EpCAM and KRT‐5), mesenchymal or myofibroblast markers (Vimentin and α‐SMA), and extracellular matrix (ECM) markers (MMP‐2 and MMP‐9) in A549 cells transfected with uc.77 or 05Rik respectively. Error bar represents biological replicates (*n* = 3). *, *P* < 0.05, **, *P* < 0.01 in comparison to cells transfected with control vector. (**F**) Western blots showing changes in protein expression of various EMT markers in A549 cells transfected with control or uc.77 or 05Rik lncRNA. The band intensity was quantified and the fold change (lncRNA/control normalized to GAPDH) was shown on the right. (**G**) Immunofluorescence images showing staining of A549 cells transfected with control or uc.77 or 05Rik lncRNA with DAPI (blue), or antibodies against vimentin (red) or E‐cad (green). Scale bar represents 100 μm.

To directly establish the causality between up‐regulation of uc.77 and 05Rik and changes in the expression of *Zeb2* and *Hoxa3* in human, we overexpressed uc.77 and 05Rik in human lung epithelial A549 cells (Fig. 5A and B) and found that increased uc.77 led to an enhanced RNA and protein expression of *Zeb2*, while 05Rik overexpression suppressed *Hoxa3* transcription (Fig. 5C, D and F). These findings are consistent with the results from our mouse model studies (Fig. 4B). Given the pivotal roles of *Zeb2* and *Hoxa3* in EMT, we also tested the effects of uc.77 and 05Rik increase on the expression of well‐known EMT markers. Expression of epithelial markers such as E‐cad, epithelial cell adhesion molecule and KRT5 was significantly downregulated in A549 cells transfected with uc.77 or 05Rik. Moreover, there was a >2‐fold increase in the expression levels of mesenchymal markers including vimentin and α‐SMA. Furthermore, ECM markers (MMP‐2 and MMP‐9) showed enhanced expression after uc.77 or 05Rik transfection (Fig. 5E). Immunoblotting and IF analyses confirmed that at the protein level there was a reduced expression of E‐cad and increased expression of vimentin, N‐cad and α‐SMA (Fig. 5F and G). As A549 cells are derived from cancerous source, we also validated our findings with primary bronchial epithelial cells isolated from healthy adults (Fig. S2A and B). Finally, A549 cells overexpressing uc.77 or 05Rik also exhibited morphological changes that are characteristic of EMT, including a more spindly and elongated cell shape and pronounced scattering of cells (Fig. S2C). Taken together, these data suggest that uc.77 and 05Rik regulate the expression of their predicted targets and are able to at least partially promote EMT.

### ZEB2 plays a critical role in uc.77‐induced EMT

**Figure 6:**
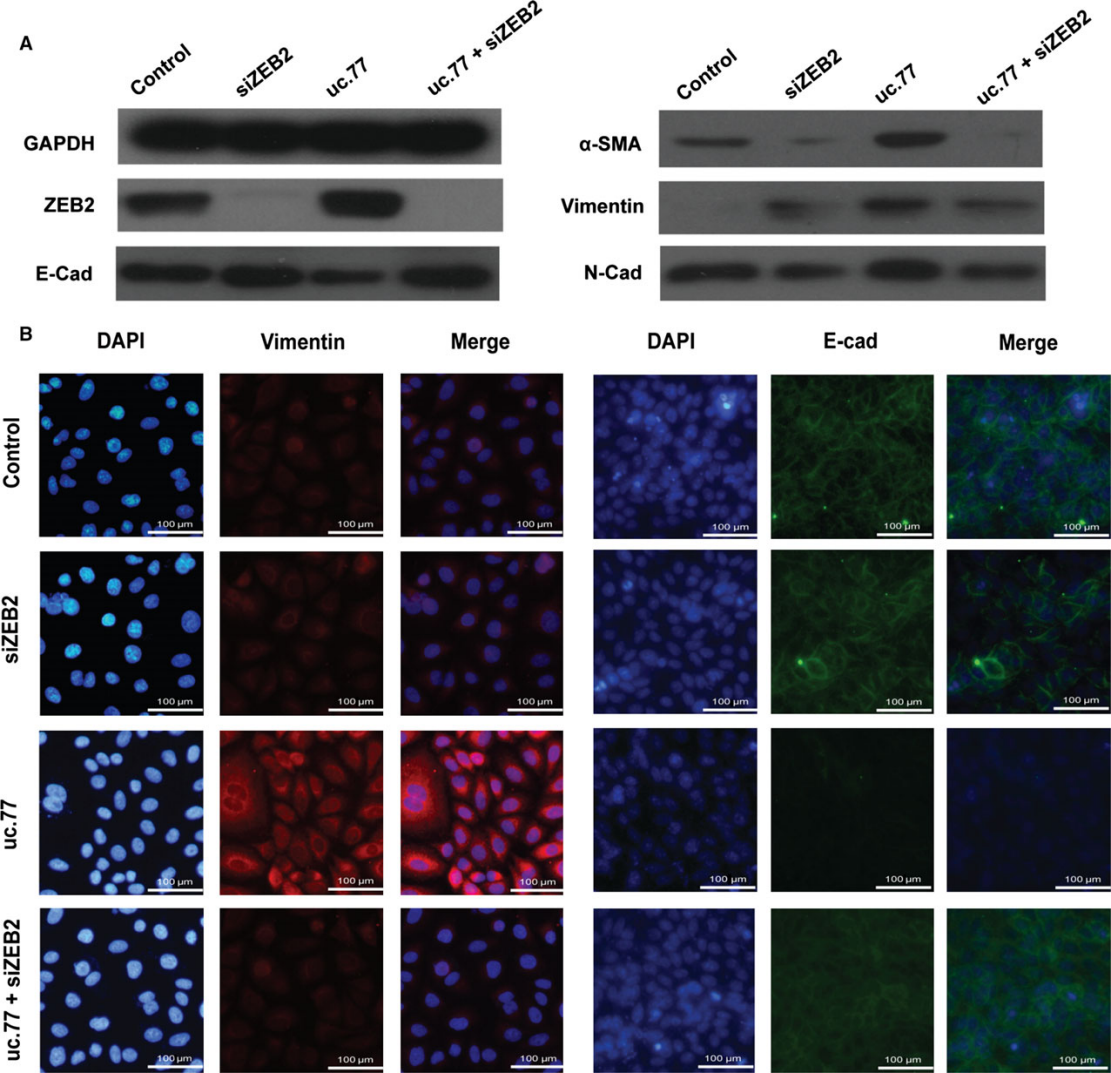
ZEB2 plays a critical role in uc.77‐induced EMT. (**A**) Western blots showing changes in protein expression of various EMT markers in A549 cells transfected with control, ZEB2 siRNA, uc.77 or ZEB2 siRNA + uc.77. (**B**) Immunofluorescence images showing staining of A549 cells transfected with control, ZEB2 siRNA, uc.77 or ZEB2 siRNA + uc.77 with DAPI (blue) or antibodies against vimentin (red) or E‐cad (green). Scale bar represents 100 μm.

Similar to microRNAs, many lncRNAs are likely to have more than one target genes and thus a pleiotropic effect. To verify that the predicted target gene is not only regulated by lncRNA, but also pivotal to the EMT phenotype induced by lncRNA, we used siRNA to knockdown ZEB2 and found that loss of ZEB2 was sufficient to reverse the onset of EMT upon uc.77 overexpression (Fig. 6A and B). Therefore, it seems that enhanced expression of ZEB2 is an indispensable part of the mechanism by which uc.77 promotes EMT during lung fibrosis.

## Discussion

In this study, we provide evidence that misregulation of lncRNAs is a prominent feature associated with the development of pulmonary fibrosis induced by paraquat in mouse. Importantly, we identified potential targets of two of the mis‐expressed lncRNAs to be crucial regulators of EMT, and demonstrated that their overexpression was sufficient to alter the transcription of target genes and partially facilitate EMT of human lung epithelial cells. Collectively, our data support a novel causal role of lncRNA in the development of EMT – a critical step in the progression of pulmonary fibrosis.

Although lncRNAs have been only recently described, they have emerged as important regulators of a wide range of biological processes including epigenetic control of gene expression. Previous studies have implicated lncRNAs in the pathogenesis of a variety of human diseases 20, and other non‐coding RNAs such as microRNAs are involved in the development of pulmonary fibrosis 15. Therefore, it is plausible that lncRNAs may similarly play an important role in this disease. Indeed, using the second‐generation lncRNA microarray, we identified 513 up‐regulated and 204 down‐regulated lncRNAs in paraquat‐induced fibrotic lung tissues compared with normal tissues by setting a threshold of fold change ≥5.0. The profound difference in lncRNA transcriptome between normal and fibrotic tissues not only imply that lncRNAs may participate in the disease initiation and progression, but also suggest that lncRNAs may be developed as valuable biomarkers for IPF in human.

Our work, together with earlier studies of lncRNA profiling using a bleomycin‐induced lung fibrosis model in rats 21, 22, represent the first investigations of lncRNAs’ function in animal models of IPF. Notably, there are a number of important distinctions between pulmonary fibrosis induced by bleomycin and paraquat in rodents 23, 24, 25. Our pathological analysis revealed that paraquat‐treated lungs exhibited prominent destruction of pro‐fibroblasts, intra‐alveolar fibrosis and obliterative fibrosis. In contrast, bleomycin treatment first induces desquamated cells and disintegrated alveoli, before interstitial fibrosis develops with fibrous thickening of the alveolar wall. Therefore, bleomycin exposure causes repetitive micro‐injuries over a period of weeks to months, while paraquat exposure results in a single acute lung injury. Moreover, unlike bleomycin, fibroblastic cells because of paraquat poisoning appear within the alveoli and do not arise from the alveolar cells themselves. Hence, although phenotypically similar, the underlying mechanisms of lung fibrosis modelled by bleomycin *versus* paraquat appear to be different. This notion is supported by the recent findings that on the transcriptome level there was a significant difference between fibrotic lung tissues from bleomycin‐ and paraquat‐treated mice 6 and by the fact that there is limited overlap between the lists of differentially expressed lncRNAs reported by us and Cao *et al*. 21. We believe that paraquat‐induced mouse model of lung fibrosis represents an important and complementary tool to fully understand IPF in human.

Interestingly, when we performed GO and KEGG pathway analysis to gain functional insights into how lncRNA might facilitate fibrosis development, a number of top enriched biological processes such as lung or epithelium development and response to wounding are closely linked to EMT. We therefore hypothesized that some of the dysregulated lncRNAs may promote lung fibrosis *via* directly targeting genes involved in EMT. Through bioinformatic analysis, we identified two of such lncRNAs, uc.77 and 05Rik. uc.77 is up‐regulated in fibrotic lung tissues and is predicted to target *Zeb2*. Zeb2 is well‐known for its important role in EMT. For example, it was shown that the complete suppression of EMT, either through genetic or pharmacological approaches, requires inhibition of Zeb2 expression 26, 27. 05Rik, also up‐regulated in fibrotic lung tissues, is predicted to control the expression of *Hoxa3*. As part of clusters A of homeobox genes, Hoxa3 acts as a ‘master’ transcription factor to affect a variety of developmental pathways. Previous studies have demonstrated that Hoxa3 can modulate tissue remodelling *via* coordinated changes in both epithelial and endothelial cell gene expression and behaviour during wound repair 28. Furthermore, altered expression of *Hoxa3* genes has been linked to pathologically induced tissue remodelling associated with tumorigenesis, as well as the process of wound repair 29.

Importantly, we validated our prediction by demonstrating that overexpression of uc.77 in cells caused an increase in *Zeb2* expression, while overexpression of 05Rik led to suppression of *Hoxa3* in human lung epithelial cells. Consistently, in paraquat‐treated fibrotic lung tissues, *Zeb2* expression was elevated while *Hoxa3* showed decreased expression. These data are consistent with the fact that both lncRNA loci are well‐conserved and suggest that the molecular function of uc.77 and 05Rik are likely to be conserved from mouse to human. It is unclear how uc.77 and 05Rik impact their target gene expression. Unlike microRNA which mainly suppresses target gene expression, gene regulation by lncRNA is more complex and poorly understood. Certain lncRNA such as HOTAIR is known to recruit transcription repressive complex to target genes to silence their expression 30. On the other hand, some lncRNA such as Evf‐2 may act to recruit specific transcription factor to enhance target gene expression 31. Future investigation is required to fully dissect the molecular mechanism by which uc.77 and 05Rik regulate the expression of EMT genes.

Lastly, we showed that overexpression of uc.77 and 05Rik was sufficient to decrease the RNA and protein expression of epithelial markers such as E‐cad while increase the expression of mesenchymal markers including vimentin and α‐SMA in both transformed and primary human lung epithelial cells. Cells expressing uc.77 and 05Rik also exhibit alterations in morphology that are typically associated with EMT. Moreover, consistent with our prediction and a critical role of ZEB2 in EMT, depletion of ZEB2 was sufficient to abolish the induction of EMT by uc.77. These findings are in agreement with recent reports demonstrating that paraquat is sufficient to induce EMT *in vitro* 32, 33. Although future investigation is required to determine whether suppressing uc.77 and 05Rik could attenuate EMT and hinder the fibrotic process, our results support the notion that mis‐regulated lncRNAs may facilitate the development of paraquat‐induced pulmonary fibrosis *via* promoting EMT.

In conclusion, our results implicate lncRNAs as important mediators in animal model of IPF and suggest that they function at least in part *via* regulating expression of genes involved in EMT. Given that the gene targets and molecular mechanisms of many lncRNAs in current study, including uc.77 and 05Rik, are conserved in human, future study is warranted to determine whether similar changes in lncRNAs transcriptome are observed in patients with IPF and assess lncRNAs as potential therapeutic targets in IPF.

## Conflicts of interest

The authors confirm that there are no conflicts of interest.

## Author contribution

Jinsong Zhang and Hao Sun designed the research study; Wei Chen collected and performed the *in vitro* experiments including plasmid construction and cell transfection. Junjie Chen and Lei Jiang performed the research of animal modelling; Jun Wang and Wenyi Qian performed cell culture experiments; Junjie Chen and Lei Jiang drafted the manuscript; Jiang Kang and Li Qiao offered critical intellectual contribution to manuscript writing. Hao Sun and Jinsong Zhang did manuscript editing and final approval. We sincerely appreciate Dr. David A. Schwartz (Department of Medicine, University of Colorado) for his help in technical optimization and critical evaluation of our experimental results.

## Supporting information


**Figure S1** (**A**) Pie chart showing 31,423 lncRNAs identified from the most authoritative databases. (**B** and **C**) Scatter plots showing the expression variation between the control and paraquat‐treated lung tissues for lncRNAs (**B**) and mRNA (**C**). LncRNAs or mRNAs above the top green line and below the bottom green line show more than threefold change.

**Figure S2** (**A**) Western blots showing changes in protein expression of various EMT markers in human bronchial epithelial cells transfected with control or uc.77 or 05Rik lncRNA. (**B**) If images showing staining of human bronchial epithelial cells transfected with control or uc.77 or 05Rik lncRNA with DAPI (blue), or antibodies against vimentin (red) or E‐cad (green). Scale bar represents 100 μm. (**C**) Representative microscopic images of A549 cells at 72 hrs after transfection with control vector or uc.77 or 05Rik. Images were taken using phase contrast microscopy (magnification: upper‐100×; lower‐200×).

**Table S1** Sequence of RT‐qPCR primers.

**Table S2** Differentially expressed lncRNAs.
