# Supplementary figures and images for: Integrated long non‐coding RNA analyses identify novel regulators of epithelial‐mesenchymal transition in the mouse model of pulmonary fibrosis
Source: J Cell Mol Med. 2016 Jan 29;20(7):1234–46. doi: 10.1111/jcmm.12783 (PMC4929291; doi:10.1111/jcmm.12783)

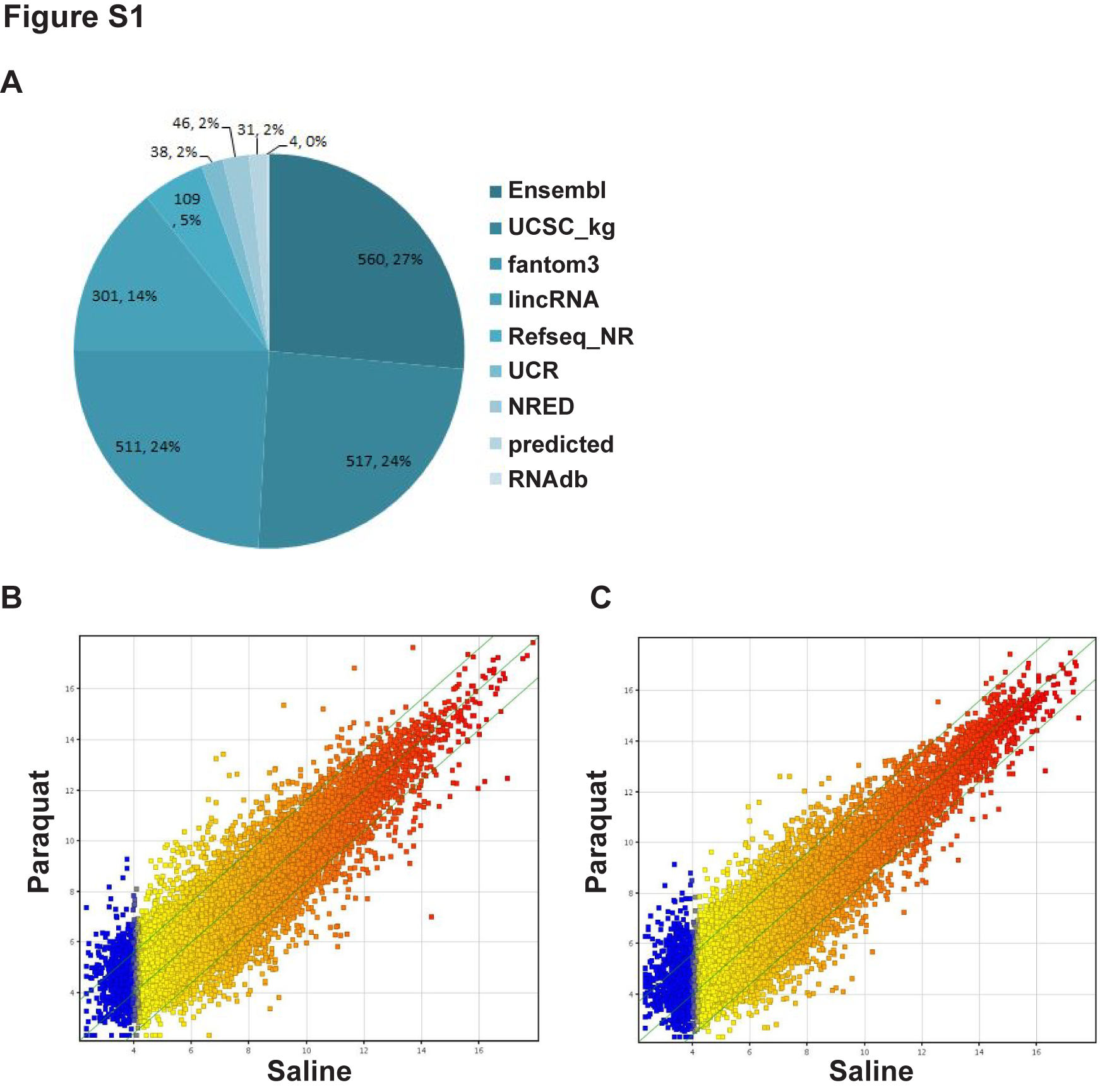

Supplement: Supplementary file 1 — Figure S1 (A) Pie chart showing 31,423 lncRNAs identified from the most authoritative databases. (B and C) Scatter plots showing the expression variation between the control and paraquat‐treated lung tissues for lncRNAs (B) and mRNA (C). LncRNAs or mRNAs above the top green line and below the bottom green line show more than threefold change. [file JCMM-20-1234-s001.tif]

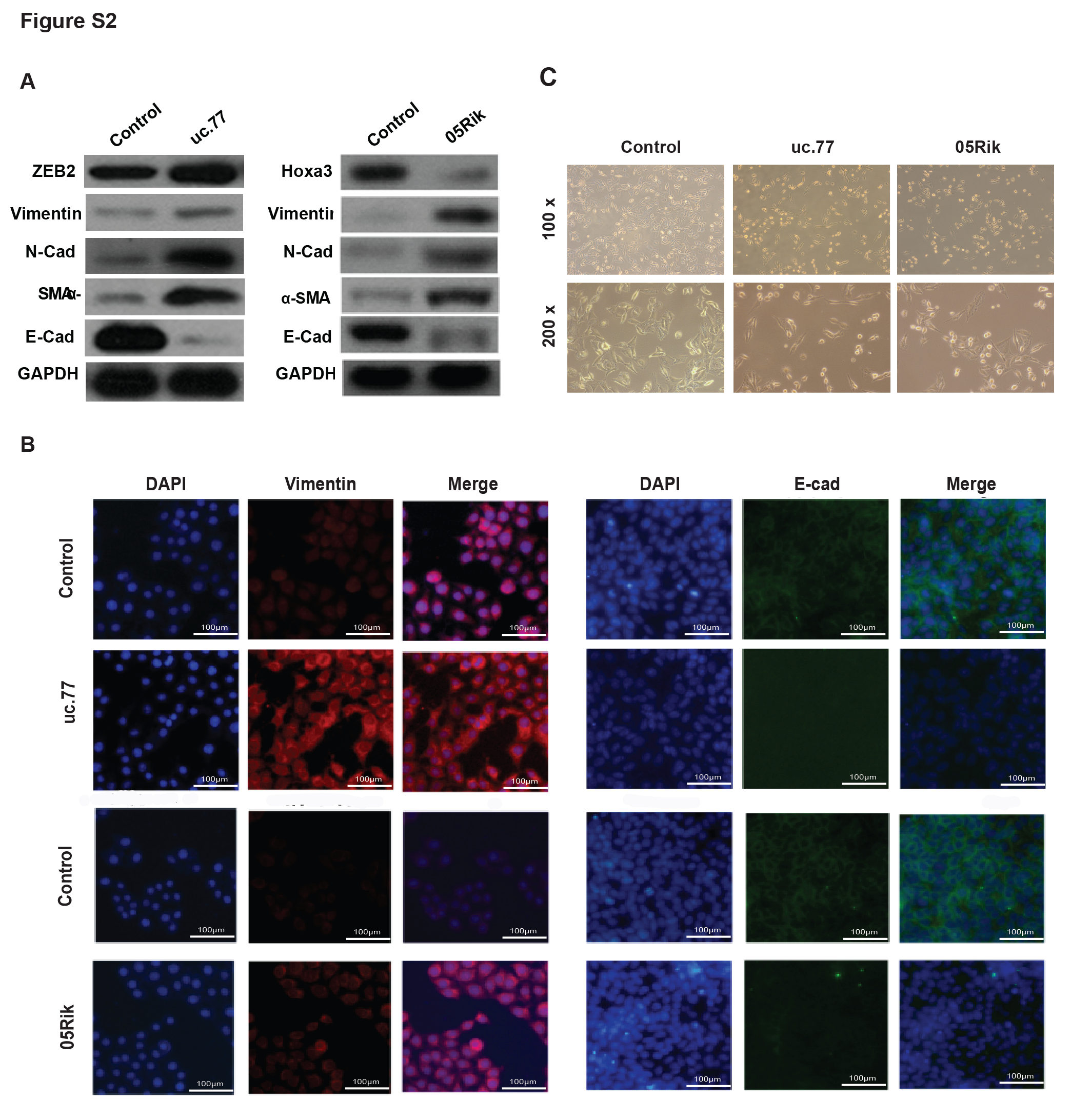

Supplement: Supplementary file 2 — Figure S2 (A) Western blots showing changes in protein expression of various EMT markers in human bronchial epithelial cells transfected with control or uc.77 or 05Rik lncRNA. (B) If images showing staining of human bronchial epithelial cells transfected with control or uc.77 or 05Rik lncRNA with DAPI (blue), or antibodies against vimentin (red) or E‐cad (green). Scale bar represents 100 μm. (C) Representative microscopic images of A549 cells at 72 hrs after transfection with control vector or uc.77 or 05Rik. Images were taken using phase contrast microscopy (magnification: upper‐100×; lower‐200×). [file JCMM-20-1234-s002.tif]
