# Supplementary material for: Integrated long non‐coding RNA analyses identify novel regulators of epithelial‐mesenchymal transition in the mouse model of pulmonary fibrosis
Source: J Cell Mol Med. 2016 Jan 29;20(7):1234–46. doi: 10.1111/jcmm.12783 (PMC4929291; doi:10.1111/jcmm.12783)
Supplement: Supplementary file 3 — Table S1 Sequence of RT‐qPCR primers. [file JCMM-20-1234-s003.docx]

**Supplementary Table 1. Sequence of RT-qPCR primers**

| LncRNAs/Gene | Primers sequence(5’ to 3’ ) | |
| --- | --- | --- |
|  | Forward | Reverse |
| GAPDH | GGTGAAGGTCGGTGTGAACG | CTCGCTCCTGGAAGATGGTG |
| UC007mmq.1 | CGACTGGAGGAGAAGAGGTG | ATTCAGACGGGCATTCAAAG |
| AK052811 | ATTGGAATGGCAGGACTTTG | GCCCAGAGCTTAGGAGGACT |
| UC008dzl.1 | CCAGAGCGGAGAGGTATCTG | GGGAATCCTCGACTTCCTTC |
| ENSMUST00000159621 | CACAGACCCCAAGATCCAGT | ACCAACAAGAAGGCATCCAG |
| 2700086A05Rik | GCAAGAAGCCCTGTTGTCTC | CGTCGTCTCTCTTCCCACTC |
| UC.77 | CTGTCACACTGCTCCCAAGA | TCAGCCAAAGATGCTTGAAA |
| UC009ktt.1 | GGTTCCATCTGCAGTGGTTT | CCCCTACCCTTCAGATCCAT |
| ENSMUST00000121776 | TGGCATCCTCCTCATCTTTC | GCCTATGCAGAGGAGTTTGG |
| UC.455- | ACCTAGGGGACCACGGTACA | CGTGATGAATAAATGCCCATC |
| BC027568 | TCCGGTACATCCCTCTTCTG | TACCCTCGCAATGAGTTTCC |
| ENSMUST00000065709 | ACCCCCTATTTGGTGGACTC | AAAGCCAAGCCTGTTTAGCA |
| UC.456 | ACCCTTTGGCTGACCTTACC | TGAGGTTTTTGGTGGTGGTT |
| ENSMUST00000119054 | TGCATGTGACATCAAGCAAA | CTCCCCTGAGGATGATGAAA |
| ENSMUST00000120952 | AGGGTCGTCTTCACTGTGCT | AAGTGGTGCCTGATGGAAAG |
| ZEB2 | CCACCAGCCCTTTAGGTGTA | CCCTTGTTCTTCTGGCTGAG |
| HOXA3 | GGCCTGCTTCTTCTGCATAC | GGCCTGCTTCTTCTGCATAC |
