# Supplementary material for: Integrated long non‐coding RNA analyses identify novel regulators of epithelial‐mesenchymal transition in the mouse model of pulmonary fibrosis
Source: J Cell Mol Med. 2016 Jan 29;20(7):1234–46. doi: 10.1111/jcmm.12783 (PMC4929291; doi:10.1111/jcmm.12783)
Supplement: Supplementary file 4 — Table S2 Differentially expressed lncRNAs. [file JCMM-20-1234-s004.docx]

| **Supplementary Table 2. Differentially expressed lncRNAs** | | | | | | | | | | |
| --- | --- | --- | --- | --- | --- | --- | --- | --- | --- | --- |
| A, Up-regulated lncRNAs | | | | | | | | | | |
| Seqname | | Source | RNA  length | | Regu-  lation | Chrom | Fold change | Raw  Control | Raw  Sample | Associated gene |
| uc007kvj.1 | | UCSC | 1387 | | Up | chr11 | 15.47 | 92.08 | 934.74 | Mrps23 |
| AK131705 | | fantom3 | 456 | | Up | chr11 | 14.77 | 150.23 | 1489.76 | Phospho1 |
| NR_028331 | | RefSeq | 473 | | Up | vhr11 | 11.90 | 116.46 | 899.06 | Zfp672 |
| NR_003546 | | RefSeq | 1637 | | Up | chr13 | 11.29 | 127.10 | 929.85 | 2410002O22Rik |
| AK016356 | | fantom3 | 712 | | Up | chr16 | 11.20 | 267.72 | 2015.30 | Dscr3 |
| AK005145 | | fantom3 | 525 | | Up | chr8 | 8.72 | 201.46 | 1142.55 | Efha2 |
| uc009opl.1 | | UCSC | 1628 | | Up | chr9 | 8.53 | 176.79 | 969.40 | 7-Sep |
| uc007vkn.1 | | UCSC | 1482 | | Up | chr15 | 8.27 | 1103.89 | 6258.13 | Sema5a |
| uc008dzl.1 | | UCSC | 586 | | Up | chr18 | 7.53 | 291.90 | 1434.07 | Epc1 |
| NR_015507 | | RefSeq | 1267 | | Up | chr1 | 6.71 | 87.92 | 354.41 | Ncl |
| U59418 | | lincRNA | 1796 | | Up | chr12 | 6.41 | 1032.02 | 4552.31 | Dio3 |
| AI551087 | | lincRNA | 446 | | Up | chr11 | 5.90 | 298.47 | 1116.31 | Ankrd36 |
| uc009ktt.1 | | UCSC | 1129 | | Up | chr8 | 5.84 | 91.66 | 314.74 | Elavl1 |
| AK006956 | | NRED | 545 | | Up | chr6 | 5.68 | 150.16 | 521.86 | Nrf1 |
| **uc.77+** | | UCR | 296 | | Up | chr2 | 5.04 | 231.28 | 718.07 | Zeb2 |
| **ENSMUST00000139055** | | Ensembl | 575 | | Up | Chr6 | 7.05 | 244.43 | 1101.14 | Hoxa3 |
| B, Down-regulated lncRNAs | | | | | | | | | | |
| Seqname | Source | | | RNA  length | Regu-  lation | Chrom | Fold change | Raw  Control | Raw  Sample | Associated gene |
| uc007hdb.1 | UCSC | | | 891 | Down | chr10 | -15.14 | 20183.46 | 1018.18 | Lyz2 |
| uc007kgm | UCSC | | | 208 | Down | chr11 | -10.89 | 7947.43 | 492.50 | Ssh2 |
| ENSMUST00000040306 | Ensembl | | | 1042 | Down | chr17 | -10.12 | 49601.20 | 4545.77 | H2-Ea-ps |
| uc008vfy.1 | UCSC | | | 2169 | Down | chr4 | -8.46 | 17123.13 | 1610.60 | D4Wsu53e |
| uc.420- | UCR | | | 233 | Down | chr11 | -7.66 | 19786.43 | 2113.88 | Ddx5 |
| uc009mqh. | UCSC | | | 1971 | Down | chr8 | -7.52 | 1265.02 | 85.83 | Itfg1 |
| uc009rwm.1 | UCSC | | | 2009 | Down | chr9 | -7.23 | 10325.13 | 1058.93 | Pdcd6ip |
| NR_033197 | RefSeq | | | 1214 | Down | chr15 | -7.03 | 28846.22 | 3456.44 | Jrk |
| uc007ism.1 | UCSC | | | 213 | Down | chr11 | -6.69 | 9276.96 | 1013.20 | Hnrnph1 |
| uc007sgh.1 | UCSC | | | 363 | Down | chr14 | -6.76 | 292.17 | 21.57 | Thoc7 |
| ENSMUST00000121455 | Ensembl | | | 2257 | Down | chr11 | -6.75 | 3185.95 | 267.46 | Ssh2 |
| uc.50- | UCR | | | 222 | Down | chr17 | -6.07 | 1885.01 | 163.69 | Srsf7 |
| uc008vzg.1 | UCSC | | | 3803 | Down | chr4 | -5.61 | 9524.59 | 1271.26 | Plekhg5 |
| NR_036615 | RefSeq_NR | | | 2752 | Down | chr17 | -5.39 | 3667.98 | 403.34 | Srsf7 |
| uc009ihm.1 | UCSC | | | 730 | Down | chr7 | -5.30 | 1817.88 | 182.64 | Crebzf |
| AK031320 | fantom3 | | | 1794 | Down | chr10 | -5.04 | 1586.47 | 165.43 | Pln |
| This list exhibit parts of the lncRNAs microarray data. 16 up-regulated and 16 down-regulated lncRNAs were shown as above. It contains the lncRNAs’ name (seqname), source, length, location in chromosome, raw intensities of each group and also their associated genes | | | | | | | | | | |
